# Analysis of the HIV-2 protease’s adaptation to various ligands: characterization of backbone asymmetry using a structural alphabet

**DOI:** 10.1038/s41598-017-18941-3

**Published:** 2018-01-15

**Authors:** Dhoha Triki, Mario Enrique Cano Contreras, Delphine Flatters, Benoit Visseaux, Diane Descamps, Anne-Claude Camproux, Leslie Regad

**Affiliations:** 1 Molécules thérapeutiques in silico (MTi), INSERM UMR-S973, Paris, France; 2 IAME, INSERM UMR 1137, Laboratoire de Virologie, Hôpital Bichat, AP-HP, Paris, France; 3 Université Paris Diderot, Sorbonne Paris Cité, Paris, France

## Abstract

The HIV-2 protease (PR2) is a homodimer of 99 residues with asymmetric assembly and binding various ligands. We propose an exhaustive study of the local structural asymmetry between the two monomers of all available PR2 structures complexed with various inhibitors using a structural alphabet approach. On average, PR2 exhibits asymmetry in 31% of its positions—i.e., exhibiting different backbone local conformations in the two monomers. This asymmetry was observed all along its structure, particularly in the elbow and flap regions. We first differentiated structural asymmetry conserved in most PR2 structures from the one specific to some PR2. Then, we explored the origin of the detected asymmetry in PR2. We localized asymmetry that could be induced by PR2’s flexibility, allowing transition from the semi-open to closed conformations and the asymmetry potentially induced by ligand binding. This latter could be important for the PR2’s adaptation to diverse ligands. Our results highlighted some differences between asymmetry of PR2 bound to darunavir and amprenavir that could explain their differences of affinity. This knowledge is critical for a better description of PR2’s recognition and adaptation to various ligands and for a better understanding of the resistance of PR2 to most PR2 inhibitors, a major antiretroviral class.

## Introduction

The human immunodeficiency virus (HIV) of type 2 is a retrovirus that was isolated in 1985 from Western African patients presenting AIDS (acquired immune deficiency syndrome) but that were HIV of type 1 (HIV-1) seronegative. The HIV of type 2 (HIV-2) therapeutic arsenal is limited compared to HIV-1. Indeed, among the antiretroviral classes targeting several viral enzymes, such as reverse transcriptase, fusion protein, integrase and protease (PR) inhibitors, HIV-2 naturally presents resistance to all non-nucleosidic inhibitors of reverse transcriptase, the fusion inhibitor and most of the protease inhibitors (PIs)^1–6^. Among the latter, the potency of FDA (Food and Drug Administration)-approved PIs for HIV-2 protease (PR2) compared to HIV-1 protease (PR1) is decreased by factors ranging from 2 to 80, resulting in only 3 usable PIs for HIV-2: saquinavir, lopinavir, and darunavir (DRV)^1,7^. Recent *in vivo* studies also showed that HIV-2 does not present a stronger virological response to the more recently available class of integrase inhibitors than previously observed response to PIs^8^, underlying the need for a third strong antiretroviral agent that will prevail against HIV-2 infection. Thus, it is still necessary to develop new molecules designed for HIV-2 today.

HIV PR is essential for hydrolysing the viral Gag and the Gag-Pol precursor polyproteins during the maturation of infectious viral particles. PR is an aspartic protease corresponding to a C2-symmetric homodimer of 99 residues in each monomer. The binding site is located at the interface between the two monomers and includes the catalytic triplet, Asp-Thr-Gly, conserved in all aspartic proteases. The PR recognizes various non-homologous substrates (Gag and Pol polyproteins) at several cleavage sites and PIs^9^. All these ligands are often asymmetric, and their binding is associated with large conformational changes resulting in a transition from a semi-open form to a closed form. How these symmetric enzymes—i.e., with two monomers exhibiting the same conformation—adjust themselves to recognize various substrates and diverse inhibitors is well described for PR1 but not for PR2. The structural asymmetry of PR1 allows the adaptation and recognition of non-homologous substrates. A comparison of six enzyme-substrate complexes of PR1 has shown that substrate binding breaks the symmetry of PR1^9,10^. Thus, to recognize and bind various asymmetric substrates, the two monomers of PR1 adopt different conformations. Moreover, PR1’s specificity for its substrates appears to be determined by an asymmetry shape rather than a particular amino acid sequence of the substrate^9,10^. In PR2, structural asymmetry has been previously detected: the two PR2 monomers exhibit slightly different orientations resulting in a molecular ‘two-fold axis’ ranging from 178.20° to 179.80° and a root mean square deviation (RMSD) ranging from 0.35 to 1.02 Å^11–14^. The largest deviations between the two monomers of the PR2 dimer have been localized in some tail, elbow and flaps regions^11–14^. A limitation of all these studies is the use of crystallographic structures with a single type of ligands without comparison of results obtained with various ligands to discriminate between ligand-induced and intrinsic asymmetry. To date, the link between structural asymmetry observed in PR2 and its capacity to bind various substrates and ligands has not been studied. Understanding the structural deformation of PR2 involved in the recognition of divers ligands is important in the design and optimization of PR2 inhibitors.

In this study, we focused on the detection of structural local asymmetry in the PR2 dimer complexed with a diversified set of ligands. To do so, we located positions exhibiting backbone structural asymmetry by using an original approach based on the HMM-SA structural alphabet (Hidden Markov Model – Structural Alphabet)^15^ to identify residues exhibiting different backbone conformations between the two PR2 chains in 19 wild-type PR2 dimers. HMM-SA was previously used to identify and characterize structural changes upon protein-protein interaction^16^ and upon ligand-binding^17^. The asymmetric positions were then classified according to their frequency in the PR2 set, allowing the differentiation of structural asymmetry observed in most PR2 dimers from the asymmetry that is specific to some dimers. According to the composition of the PR2 set, several reasons could explain the observed structural asymmetry: the intrinsic flexibility of PR2, PR2’s dimerization and its ligand binding. To differentiate asymmetry resulting from PR2 flexibility, we performed matching of the observed structural asymmetry with PR2’s flexible positions. We then compared the location of asymmetric positions with the ligand-binding pocket of PR2 to highlight structural asymmetry potentially induced by ligand binding. This asymmetry is important for PR2’s adaptation to the ligand and for ligand recognition. We also localized structural asymmetry positioned at the PR2 interface (the region where the two monomers interact) to identify structural asymmetry that could result from PR2 dimerization. Our results should improve the understanding of structural changes of PR2 and its adjustments to recognize and bind various inhibitors and the understanding of PR2 determinants to explain its resistance against some FDA-approved drugs.

## Materials and Methods

### PR2 set presentation

The PR2 dimer set is composed of 19  crystallographic structures of wild-type PR2 extracted from the Protein Data Bank (PDB)^18^. These structures have good resolution, ranging from 1.18 to 3 Å. All these PR2 structures present the same amino acid sequence except eight that contain the mutation K57L (an experimental mutation introduced to help the crystallographic process). This PR2 set contains one unbound PR2 dimer—i.e., not complexed to a ligand—and 18 dimers complexed with various ligands (Table S1).

**Figure 1:**
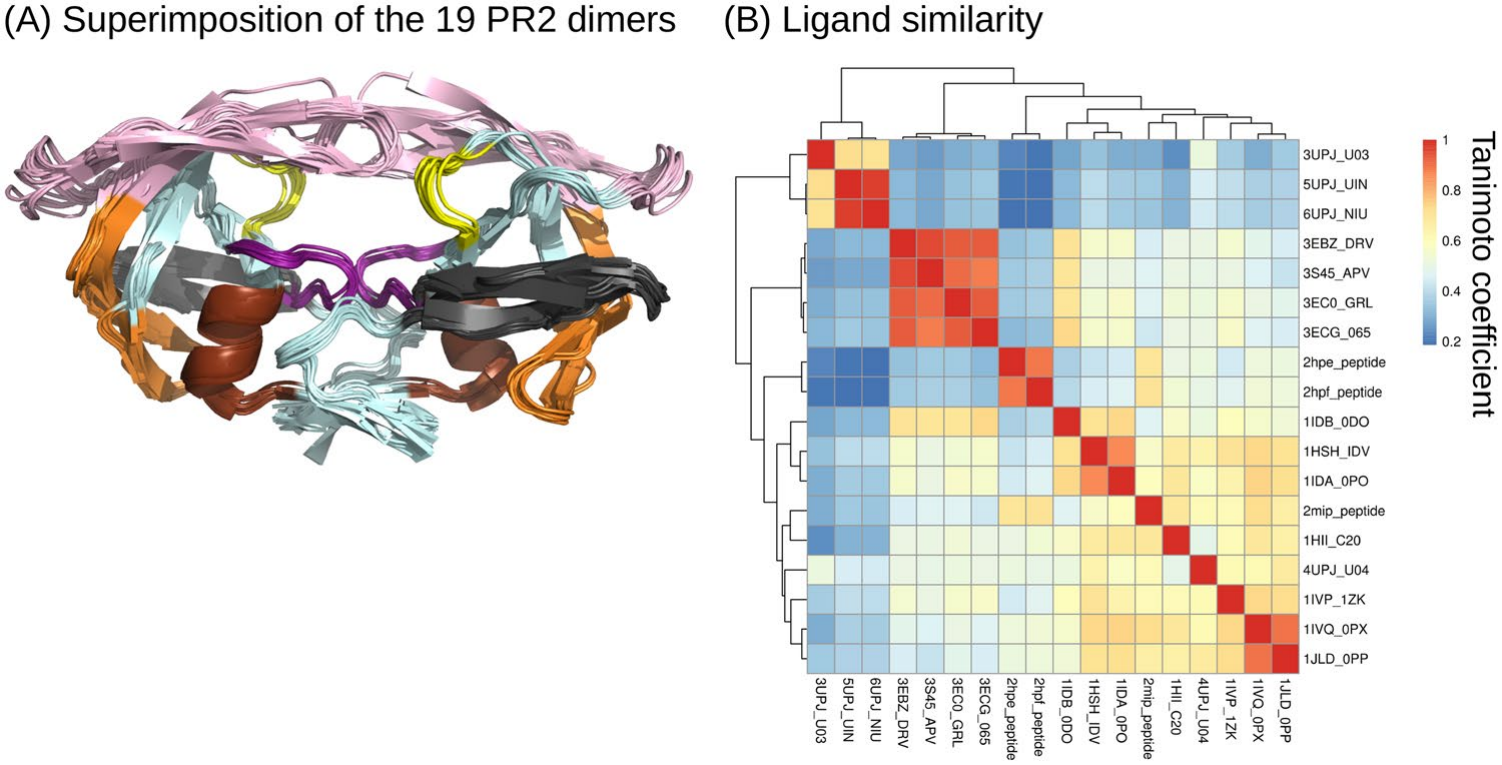
Presentation of the PR2 set. (**A**) Superimposition of the 19 PR2 dimers extracted from the PDB (coloured in sky blue). The proteins are displayed as cartoons and coloured according to the functional and structural regions defined by Sadiq and De Fabritiis, 2010^24^: The fulcrum (10–23) is coloured grey, the catalytic site (24–30) is coloured purple, the elbows (37-42) and flaps (43–58) are coloured pink, the cantilever (59–72) is coloured orange, the wall is coloured yellow (80–83) and the α-helix (87–95) is coloured brown. (**B**) Illustration of ligand similarity obtained using a Tanimoto coefficient matrix. Ligands were named using the following name: “PDB code”_“HETATM code”. A hierarchical classification of the ligands was computed using the Jaccard distance (1 – Tanimoto coefficient) and the ward aggregation method using the hclust function of R software^23^. The closer the box is to the red, the more similar the pair of ligands. Conversely, the closer the box is to blue, the less similar the pair of ligands.

### Detection and quantification of the PR2 asymmetry using the HMM-SA structural alphabet

#### Definition of structural asymmetric positions

**Figure 2:**
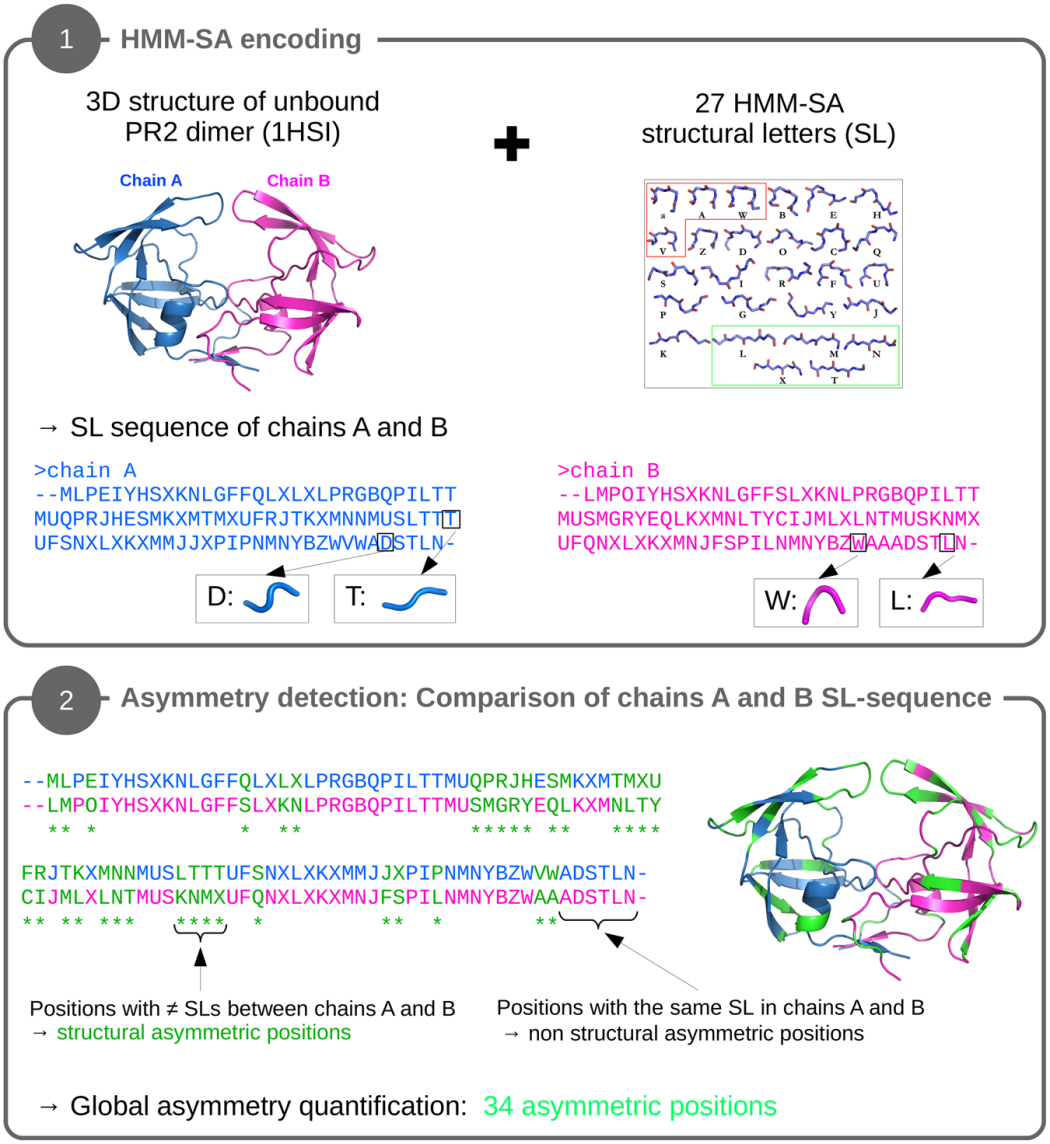
Presentation of the SA-based protocol to extract structural asymmetric positions. First the tri-dimensional structure of the unbound PR2 (99 residues, PDB code: 1HSI) is simplified into a sequence of 96 structural letters using the HMM-SA structural alphabet^15^. This results in two structural letter sequences associated with each chain, where each structural letter describes the geometry of a four-residue fragment. The geometry of four structural letters (T, D, W, and L) extracted from chains A and B is illustrated. Then the structural-letter sequence of the two chains is compared to locate the asymmetric positions, defined as positions exhibiting different structural letters in chains A and B.

We defined a position as asymmetric in a PR2 dimer if it exhibits different backbone local conformations in the two monomers (chains A and B) using the protocol presenting in Fig. 2. To determine the local conformation of all positions of each PR2 monomer, we used the HMM-SA structural alphabet^15^. HMM-SA is a library of 27 structural prototypes of four consecutive Cα, called structural letters, established after a geometric classification of overlapping protein four-Cα fragments using the hidden Markov model^15^. HMM-SA is an effective and relevant tool for the study of protein structures^19^, protein deformations^16^, and protein loop conformations^20^, and for extracting structural motifs from protein loops^17,20^.

First, HMM-SA was used to simplify the three-dimensional structure of each PR2 monomer (99 residues) into sequences of 96 structural letters, in which each structural letter describes the local geometry of each four-Cα fragment (*i-2*, *i-1*, *i*, and *i* + *1*) and is assigned to the third residue (*i*) of the four-Cα fragment. This simplification consisted of determining the geometrically closest structural letter of each overlapping four-Cα fragment of the protein structure using the Viterbi algorithm and took into account both the structural similarity of the fragments with the 27 structural letters and the preferred transitions between structural letters. Second, we compared the structural-letter sequences of the two monomers for each PR2 to localize the asymmetric positions. An asymmetric position was defined as a position exhibiting different structural letters between the two monomers A and B.

#### Parameters used to quantify the structural asymmetry of the PR2 set

Several parameters were used to quantify the global and local structural asymmetry of the PR2 dimer set. First, the global structural asymmetry of each PR2 dimer was evaluated by determining the number of asymmetric positions observed between its two chains A and B. Second, the structural asymmetry of all PR2 positions was evaluated for each PR2 dimer using the magnitude of asymmetry. We quantified the magnitude of asymmetry for each position *i* in each dimer by determining the RMSD between the atomic positions of the structural letters observed in chains A and B at position *i*, noted *RMSD*_*A/B*_*(i)*. The *RMSD*_*A/B*_ between two structural letters has previously been computed from 500 fragment pairs randomly chosen in the two structural letters^15^. According to the *RMSD*_*A/B*_ values, we classified the structural asymmetric positions. A position having an *RMSD*_*A/B*_ smaller than 0.5 Å corresponds to a position exhibiting structural asymmetry with small magnitude. A position with an *RMSD*_*A/B*_ value ranging from 0.5 to 1 Å is defined as a position exhibiting asymmetry with medium magnitude. A position with an *RMSD*_*A/B*_ value larger than 1  Å corresponds to a position exhibiting asymmetry with large magnitude. The average magnitude of particular asymmetric positions was compared using a Wilcoxon test.

Finally, to evaluate whether structural asymmetry was observed frequently or rarely (due to a particular ligand or particular experimental condition) in the PR2 set, we computed, for each position, the number of PR2 dimers exhibiting the position as asymmetric, and we named this asymmetry occurrence (*AO)*. The statistical significance of the *AO* value of a position *i* was determined using the overrepresentation p-value of any *AO*, denoted *pvalue*^*AO*^. The *pvalue*^*AO*^ was estimated as the probability that the expected *AO* computed in a random set, denoted *AO*^*random*^*(i)*, is higher than *AO(i)* using a set of 2000 generated random sets (Equation 1 and Supplementary Note 1).

1 $$pvalu{e}^{AO}(i)=p[A{O}^{random}(i) > AO(i)=n\{A{O}^{random}(i) > AO(i)\}/{n}_{simu}$$

where *n* {*AO*^*random*^*(i)*>*AO(i)*} is the number of simulations where *AO*^*random*^*(i)* is higher than *AO(i)*, and *n*_*simu*_ is the number of simulations.

An asymmetric position was considered statistically overrepresented if its *pvalue*^*AO*^ was below a threshold of 0.0005 as determined using the Bonferroni adjustment to consider multiple tests (0.05/96 positions). The repartition of overrepresented asymmetric positions in different regions of PR2 was assessed using the chi-square test.

### Analysis of putative structural asymmetry origins

To assess the putative origin explaining the detected structural backbone asymmetry in PR2, we crossed the structural asymmetry parameter with other parameters. We analysed structural asymmetry as a function of the experimental conditions associated with each PR2 dimer structure to highlight structural asymmetry that could be a bias linked to experimental methodology. We also localized the structural asymmetric positions as a function of the ligand-binding pocket to identify asymmetry that may be induced by ligand binding and may be involved in recognition of the ligands and as a function of the PR2 interface to highlight asymmetry that may be induced by PR2 dimerization. We also differentiated asymmetries located in rigid positions from those observed in flexible positions to identify any asymmetry induced by the intrinsic flexibility of PR2.

#### Determination of the experimental information about each PR2 structure

We extracted two parameters related to the crystallography experience from each PDB file. The first parameter is the resolution of the structure measuring the resolvability in the electron density map of a molecule. The second parameter is the space group where the structure was determined. It describes how identical objects can be arranged in orderly arrays in an infinite three-dimensional lattice network—i.e., the symmetry of a crystal. The 19 PR2 structures were crystallized in six different space groups (Table S1). Several PR2 structures were crystallized in the same space group, such as 3S45, 3EBZ, 3ECG, and 3EC0 (PDB codes) crystallized in the “C121” space group. In contrast, some space groups areunique, for example, 2HPF (PDB code) is the only one PR2 crystallized in the “P6_5_” space group.

For each structure, we also identified the ligand complexed with the PR2 dimer (Table S1). These ligands were classified according to their type: three FDA-approved drugs (DRV, amprenavir (APV), and indinavir), two drug derivates (GRL and 065), four experimental molecules, three synthetic analogue inhibitors, three peptides (including two non-determined ones), and four peptidomimetic molecules). The chemical diversity of these ligands was assessed using Tanimoto coefficients computed on all pairwise ligands using MACCS fingerprint^21^ in the Openbabel programme^22^. Ligand similarity was illustrated using a Tanimoto coefficient matrix, Fig. 1B. The closer the Tanimoto score is to 1, the more similar the ligands are. In addition, a hierarchical classification of the ligands was computed using the Jaccard distance (1 – Tanimoto coefficient) and the ward aggregation method using the hclust function of R software^23^.

#### Extraction of the structural and functional regions of PR2

To determine the origin of the PR2 structural asymmetry, we crossed the localization of asymmetric positions with the localization of structural and functional regions of PR2. First, the structural and functional regions were defined using the same limit as in previous work^24^ (Fig. 1A).

Then, we determined the secondary structure of PR2 residues. To do so, the secondary structure of all residues of the PR2 structure corresponding to PDB code 3S45, extracted from the PDBsum database^25^, was used as an arbitrary reference.

**Figure 3:**
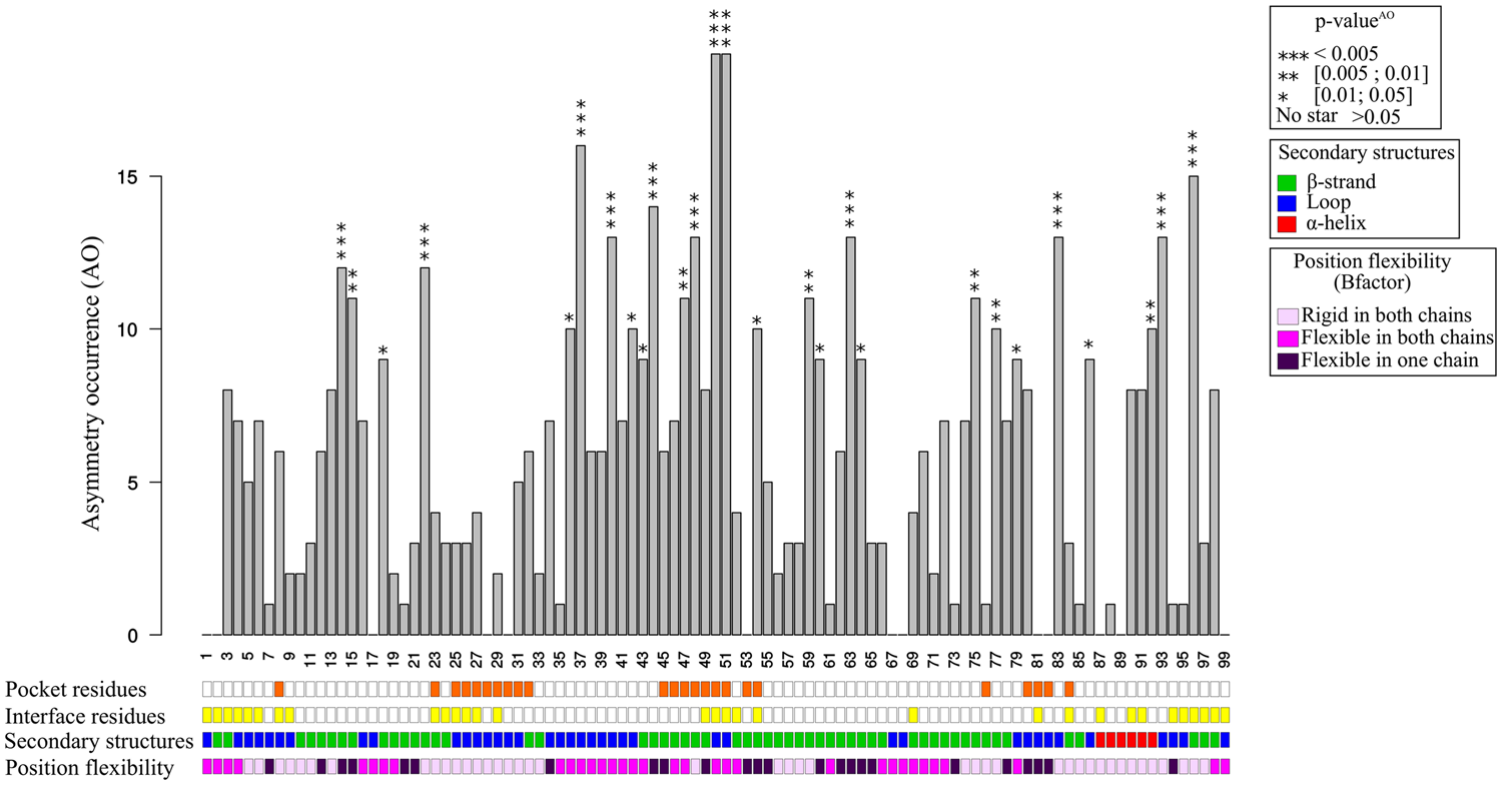
Asymmetry occurrence (AO) for the 99 PR2 positions. For each position, stars indicate the overrepresented positions with different significant thresholds. The box lines located below the graphic provide a description of PR2 positions. The first line indicates positions involved in the consensus pocket of PR2 (orange boxes), the second indicates positions involved in the PR2 interface (yellow boxes), the third indicates the secondary structures of the 99 positions, and the fourth differentiates rigid and flexible positions defined using B-factor parameter.

The ligand-binding pocket was extracted from the 18 structures of bound PR2 dimers using the proximity approach^26–28^ by determining the PR2 atoms situated at less than 4.5 Å from the co-crystallized ligand, resulting in a set of 18 binding pockets. Then, the global pocket residues were defined as residues involved in a ligand-binding pocket in at least one PR2 dimer, resulting in a consensus pocket containing 24 pocket residues (Fig. 3).

The PR2 interface was determined by extracting residues with differences in the accessible surface area (ASA) of more than 5 Å^2^ in the PR2 in dimer form—i.e., in structures that contains the two monomers—and in monomer form—i.e., in structures that contain only one monomer (Supplementary Note 2). The global PR2 interface was finally defined as the 28 residues involved in the interface in at least 80% of all PR2 dimers (Fig. 3 and Supplementary Note 2).

#### Extraction of flexible and rigid positions of PR2

The rigid and flexible positions of PR2 were determined using normalized B-factor values^29^ (temperature factor/atomic displacement factor, denoted *B*_*norm*_) extracted from the PDB files (Supplementary Note 3). This B-factor value reflects the degree of isotropic smearing of the electron density around its center^30^.

For a PR2 position, we computed its average *B*_*norm*_ value using the *B*_*norm*_ values of residues at this position in the 19 PR2 structures. A flexible position is defined as a position with an average *B*_*norm*_ higher than 0 in either chain A or chain B or in both. A rigid position is defined as a position with an average *B*_*norm*_ smaller than 0 in both chains A and B. The comparison of the structural asymmetry, defined in terms of *AO*, of flexible and rigid positions was performed using t-tests.

## Results

### Characterization of the global structural asymmetry of PR2 dimers

**Figure 4:**
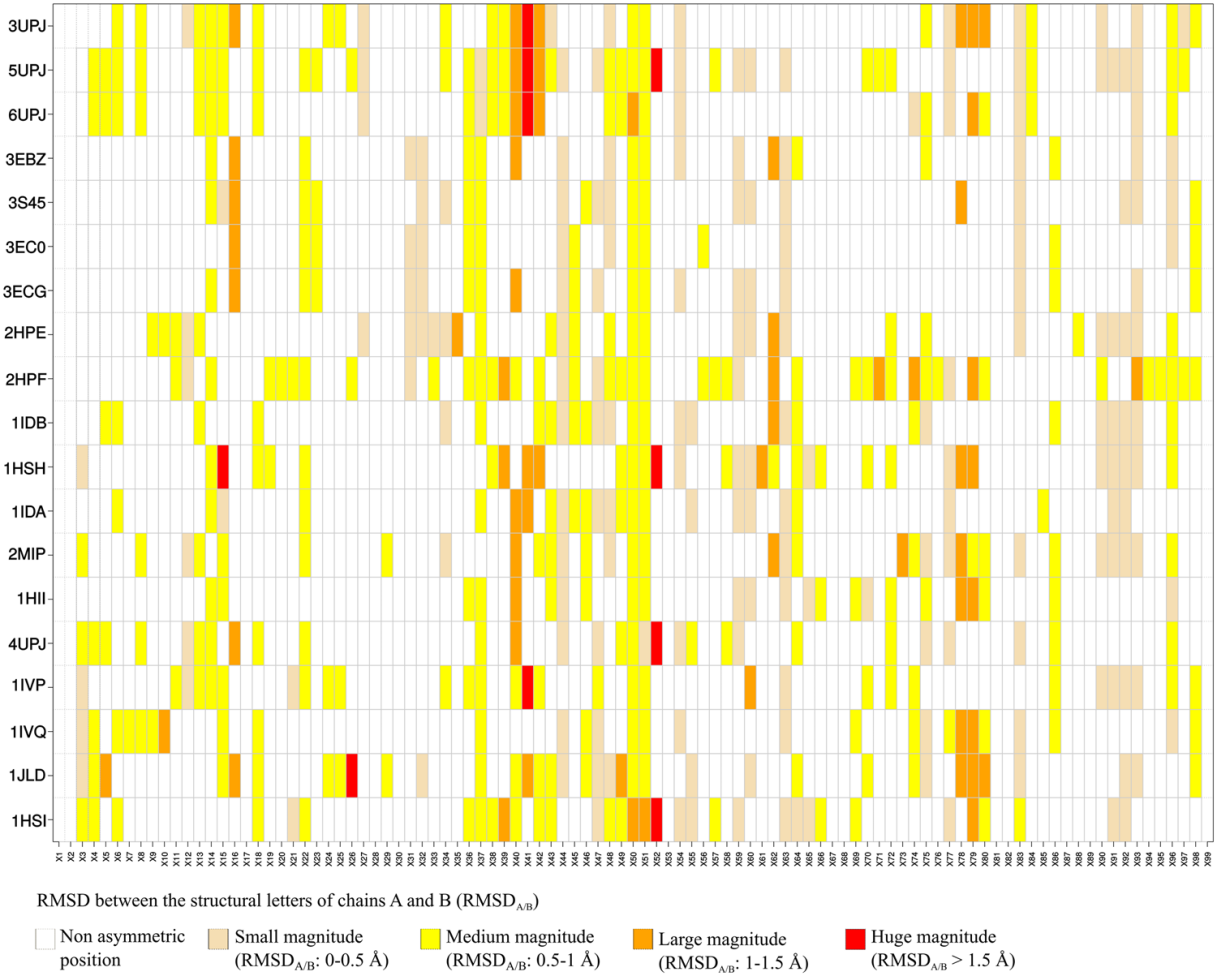
Localization of structural asymmetric positions in the 19 PR2 dimers. The PR2 dimers are presented in rows and the 99 positions in columns. Asymmetric positions are coloured according to the asymmetry magnitude quantified as the RMSD between the structural letter at a given position in chains A and B, noted RMSD_A/B_. The more the position is coloured in red, the greater the magnitude of the structural asymmetry. The PR2 dimers are ranked according to the similarity of co-crystallized ligands computed using Tanimoto coefficients.

**Figure 5:**
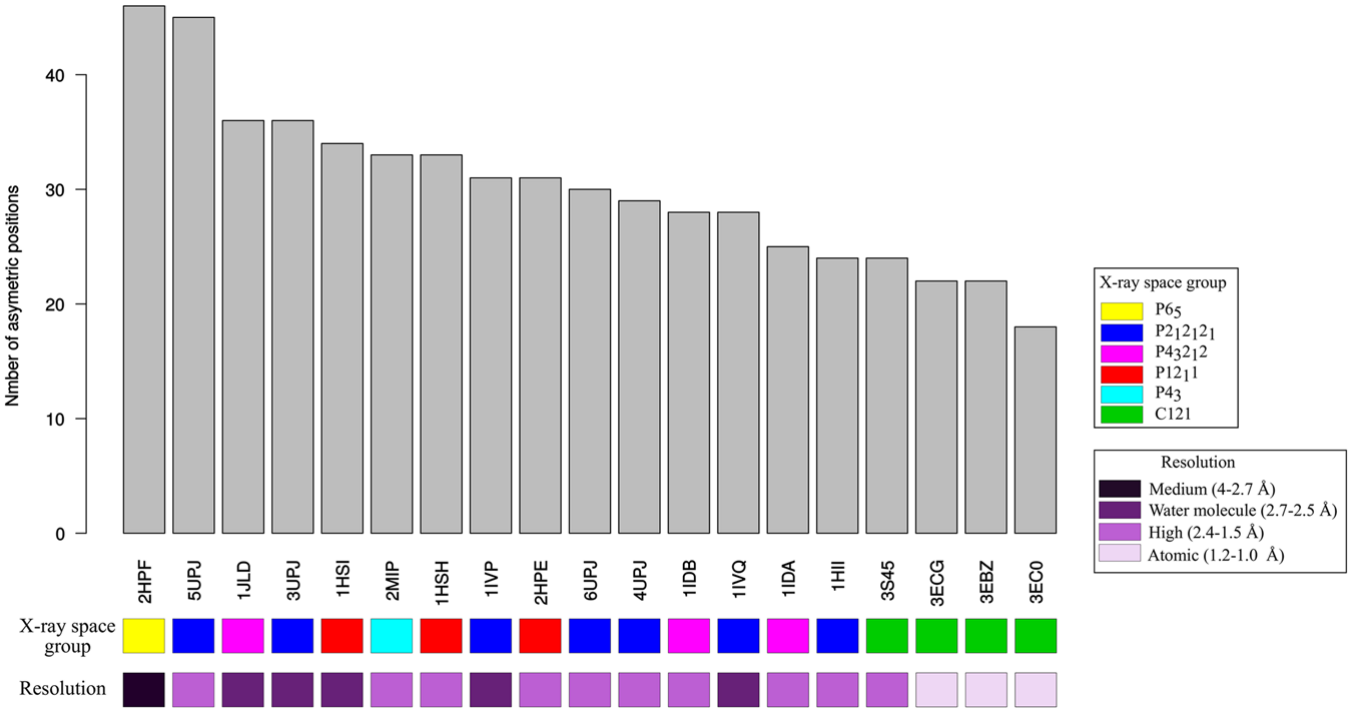
Number of asymmetric positions in the 19 PR2 structures. The PR2 dimers are ranked in decreasing order according to their global asymmetry—i.e. their number of asymmetric positions. The two boxes below the graphics provide a PR2 structure’s description by indicating its crystallographic space group and its resolution (in Å).

We determined and localized the structural asymmetric positions in the 19 PR2 dimer structures complexed with various ligands (n = 18) and in unbound form (n = 1). For each dimer, we compared the residues’ local conformations in both chains simplified into the HMM-SA space (Fig. 2). In a dimer, an asymmetric position was defined as a position exhibiting different backbone local conformations—i.e., structural letters—in each chain. Figure 4 highlights the structural asymmetric positions of each PR2 dimer. We observed that all PR2 dimers contain structural asymmetric positions located along the PR2 sequence and structure. To quantify the global asymmetry of the PR2 set, we computed the number of asymmetric positions in each PR2 dimer (Fig. 5). On average, a PR2 dimer exhibits 30 (±7) asymmetric positions (31% of its amino-acid sequence), ranging from 18 to a maximum of 46 asymmetric positions for the PR2 structures having the best (1.3 Å, PDB code: 3EC0) and the worst (3 Å, PDB code: 2HPF) resolution, respectively. Among the 19 PR2 structures, the number of asymmetric positions in a PR2 structure is not correlated with its resolution ( Pearson correlation coefficient= −0.15 and p-value = 0.54), even if the three PR2 structures with the best resolution are less asymmetric—i.e., they exhibit lower numbers of asymmetric positions. In addition, Fig. 5 shows that there is no link between the crystallographic space group, which defines the symmetry of the crystal and the global asymmetry of the PR2. Indeed, amongst the PR2 structures crystallized in space group P2_1_2_1_2_1_, some contain many asymmetric positions, such as structure 5UPJ (PDB code) with 45 asymmetric positions, while others have fewer, such as structure 1HII (PDB code) with 24 asymmetric positions. Thus, it seems that the structural asymmetry of a protein is the result of a combination of several parameters: the experimental conditions, the ligand-binding, and intrinsic properties of the PR2.

### Characterization of the structural asymmetry of each PR2 position

To analyse in more detail the structural asymmetric positions, we quantified the magnitude of the structural asymmetry by computing, for each position, the RMSD between the structural letters observed in chains A and B (*RMSD*_*A/B*_), as shown in Fig. 4. An asymmetric position has, on average, a medium magnitude quantified by an *RMSD*_*A/B*_ of 0.6 Å (±0.28). However, 12% of asymmetric positions exhibited asymmetry with a large magnitude (*RMSD*_*A/B*_ higher than 1 Å), such as positions 40 and 79 (Fig. 4). In contrast, 33% exhibited asymmetry of small magnitude with *RMSD*_*A/B*_ below 0.5 Å (Fig. 4), such as positions 44, 47, 48.

We then quantified the structural asymmetry of each PR2 position by computing its asymmetry occurrence (*AO*) (Fig. 3). Ten positions (17, 18, 30, 53, 67, 68, 81, 82, 87, and 89) were detected as non-asymmetric in any PR2 structure, while two positions (50 and 51) are asymmetric in all PR2 dimers. On average, a position exhibits an *AO* value of 6 (±4.6), meaning that the position was detected as asymmetric in six PR2 dimers. We noted that flexible and rigid positions, defined using B-factor values, exhibit the same average *AO* (t-test p-value = 0.8), meaning they exhibit similar asymmetry. This indicates that the observed asymmetries do not result only from the intrinsic flexibility of PR2. To analyse the statistical significance of the *AO* value for each position, we computed their *pvalue*^*AO*^. This parameter allowed us to distinguish two types of asymmetric positions: those overrepresented and observed in many PR2 dimers and those not overrepresented in the PR2 set. Most PR2 positions (72%) are non-overrepresented asymmetric positions, meaning that they are asymmetric in few PR2 dimers. Some of them seem to be specific to some crystal conditions or ligands. For example, PR2 dimers complexed with the similar ligands UIN, U03 and NIU (HETATM code, Tanimoto coefficient = 0.80 ± 0.14, Fig. 1B) and PR2 complexed with a peptide are the only structures exhibiting asymmetry at position 27. PR2 dimers complexed with DRV or with its derivates (similar ligands exhibiting a Tanimoto coefficient of 0.93 ± 0.001, Fig. 1B) and crystallized in the C121 space group do not exhibit structural asymmetry at positions 38, 39, 42, 43 and 49.

**Figure 6:**
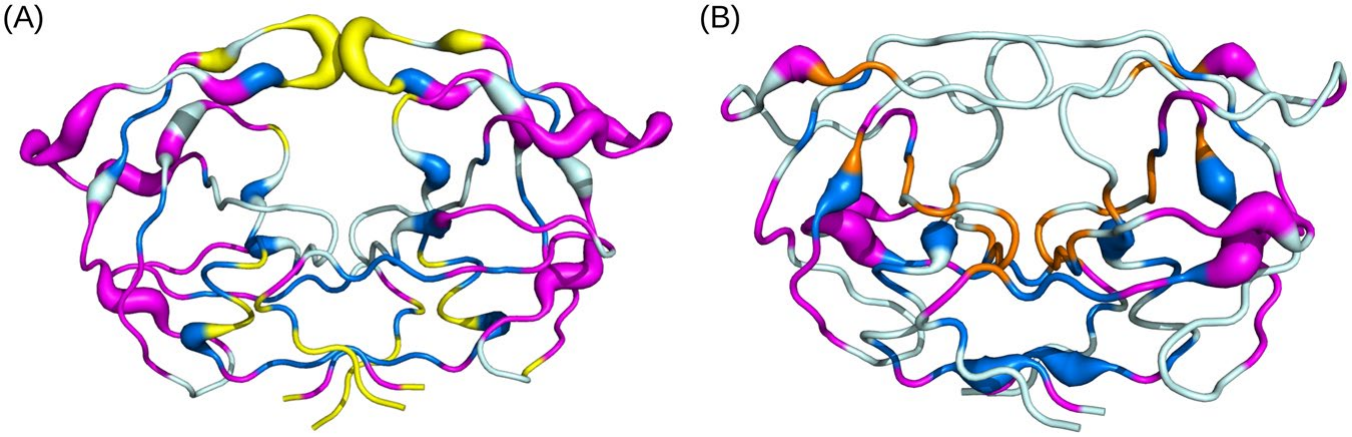
Localization of structural asymmetric positions onto two PR2 structures: (**A**) the unbound PR2 structure (PDB code: 1HSI) and (**B**) bound PR2 dimer complexed with APV (PDB code: 3S45). PR2 structures are coloured in sky blue and displayed in putty cartoon mode where large diameter indicates the overrepresented asymmetric positions. (**A**) Asymmetric positions located in the PR2 interface are coloured yellow. Rigid asymmetric positions located outside of the interface are coloured blue. Flexible asymmetric positions outside of the interface are coloured magenta. (**B**) Localization of bound-specific asymmetric positions are coloured according to their location relative to the binding site. Bound-specific asymmetric positions located in the binding pocket are coloured orange. Bound-specific asymmetries outside of the binding pocket are coloured blue if they are rigid positions (in terms of B-factor) or magenta if they are flexible positions (in terms of B-factor).

The PR2 dimer set contains 27 statistically overrepresented asymmetric positions (28%) along the whole PR2 sequence (Fig. 3). To assess the origin that can explain this asymmetry, we studied the localization of these overrepresented asymmetric positions and more particularly their matching with flexible positions defined as positions exhibiting a positive *B*_*norm*_ value. Figure 6A presents the three-dimensional structure of an unbound PR2 dimer (PDB code: 1HSI) where the overrepresented asymmetric positions have been located. Some PR2 regions exhibit a high concentration of asymmetric positions, such as the flexible regions composing the elbow (a loop) and the beginning of the flap (a β-strand), which contain 37% of all overrepresented asymmetric positions (Figs 3 and 6A). Overrepresented positions are not privileged among flexible positions (chi-square test p-value = 0.18, Fig. 3) or among some secondary structures (chi-square test p-value = 0.82; α-helix and β-strand versus in loop conformations). Moreover, the magnitude of structural asymmetry in overrepresented positions is higher than that observed in non-overrepresented positions (Wilcoxon test p-value = 0.04).

#### Characterization of structural asymmetry induced by PR2 dimerization

To highlight the structural asymmetry in PR2 induced by its dimerization, we crossed the location of asymmetric positions with the PR2 interface composed of 28 residues (Material and Methods). Except for two positions (81 and 87), these interface positions exhibit structural asymmetry (Figs 3 and 6). These asymmetric positions located at the PR2 interface correspond to both rigid and flexible positions, meaning that one part of this asymmetry seems to be induced by the interaction between the two monomers, and another part is explained by both PR2 dimerization and intrinsic flexibility. Four of these asymmetric positions are overrepresented (50, 51, 54, and 96, Fig. 3). For example, positions 50 and 51 are asymmetric in all PR2 structures but the magnitude of the structural asymmetry in these residues between both chains was higher in the unbound PR2 (*RMSD*_*A/B*_ higher than 1 Å) than in bound dimers (average *RMSD*_*A/B*_ are of 0.67 Å (±0.08) and 0.83 Å (±0.09), for positions 50 and 51 respectively; Fig. 4). We observed that residues 50 and 51 in both chains of bound forms are less flexible—i.e. they exhibit smaller *B*_*norm*_ values than in the unbound (*B*_*norm*_ value is ranking −1.41 to 2.15 Å versus 2.5 Å). This difference in both their asymmetry magnitude and flexibility is explained by a hydrogen bond mediated by a water molecule between these two residues in the bound dimer only^14^. Thus, the decrease of structural asymmetry in bound forms relative to unbound form could be linked to their reduced flexibility. Other asymmetric positions located at the interface are specific to some PR2 structures, suggesting that some portions of structural asymmetry located at the interface are affected by the experimental conditions (space group and resolution) or by ligand binding.

#### Characterization of structural asymmetry observed only in bound forms

To highlight structural asymmetry induced by ligand binding, we compared the structural asymmetry of bound PR2—i.e., complexed with a ligand—with that of unbound PR2—i.e., not complexed with a ligand. Indeed, the structural asymmetry observed in the latter PR2 dimer cannot result from ligand binding. Thus, we supposed that positions detected as asymmetric in a bound PR2 dimer but not in the unbound PR2 dimer result from ligand binding. The PR2 set contains only one unbound PR2, preventing us from testing the robustness of the observed results. However, this structure exhibits good resolution (2.5 Å) and is not crystallized in a particular crystallographic space group (P12_1_1 space group of two bound PR2 structures). In the PR2 dimer set, 53 positions were detected as asymmetric in at least one bound PR2 dimer but not in the unbound dimer, and these were named bound-specific asymmetric positions. Figure 6B localizes these positions on the three-dimensional structure of the PR2 dimer complexed with APV. Most of them (83%) are not overrepresented, meaning they are present in few structures and indicating that they are specific to some PDB files. We then analysed the matching between these bound-specific asymmetric positions and the ligand-binding pocket of PR2 (see Material and Method for the pocket determination) and the flexible positions determined using B-factor values. Among them, twelve (8, 23, 25–27, 29, 31, 32, 45, 46, 76, and 84) were located in the binding pocket (Fig. 3). Except positions 45 and 46, all these pocket asymmetric positions are rigid ones. All these observations indicate that the structural asymmetry at these positions results from ligand binding. As PR2 dimers are complexed with different ligands, this suggests a link between the detected structural asymmetry and the ligand type—i.e., some structural asymmetric positions seem to be specific to some ligands and are involved in the ligand’s recognition. For example, only six PR2 dimers exhibit structural asymmetry at position 8: three of them are bound to 4-hydroxycoumarin derivates (PDB codes: 3UPJ, 5UPJ, and 6UPJ). These three structures are also the only ones that have a structural asymmetry at position 84. Thus, binding pocket positions 8 and 84 can be involved in the specific recognition of ligands derived from 4-hydroxycoumarin. In the same way, we suggest that positions 23 and 32 could be of importance for the recognition of DRV and its derivative as they are asymmetric in most of the PR2 dimers complexed with DRV (PDB code: 3EBZ), and the two DRV derivatives (PDB codes: 3EC0 and 3ECG), Fig. 4.

Among the remaining bound-specific asymmetric positions, 41 are not inside the binding pocket; 22 of them correspond to flexible positions and, thus, are possibly induced by the intrinsic flexibility of PR2. They are mainly located in the fulcrum, flaps and cantilever. The 19 remaining asymmetric positions are rigid and were concentrated in the α-helix, the end of flaps, and the beginning of the fulcrum. Excepting four (56, 75, 93, and 96), these asymmetric positions are not overrepresented. According to these results, this asymmetry is likely related to long-range effects of the binding of some ligands.

### Putative structural asymmetry induced in the decreased susceptibility of APV for PR2

We studied with particular interest the structural differences between PR2 structures bound to APV (PDB code: 3S45) versus DRV (PDB code: 3EBZ). Indeed, even if APV and DRV are very similar molecules, as only a fluor group has been introduced into DRV comparing to APV (Tanimoto coefficient = 0.95), these two FDA-approved drugs present totally different efficiencies against PR2 with Ki values at 4.4 and 0.17 nM for DRV and APV, respectively. Comparing their asymmetry profile can bring knowledge about the difference of affinity between these two drugs against the PR2. The advantage of these structures is that they are crystallized in the same space group with close resolution (Table S1 and Fig. 4) that allows minimizing the structural differences explained by experimental conditions. Comparison of their local structural asymmetry showed that the dimers complexed with the two drugs exhibit globally similar asymmetry profiles with 70% of asymmetric positions in common. However, some differences are observed, particularly at positions 15, 23, 34, 46, 78, and 92 (Fig. 4): all are asymmetric in the PR2 bound to APV but not in the PR2 complexed with DRV. Inversely, positions 31 and 75 are detected as asymmetric in the PR2 bound to DRV but not in the PR2 complexed with APV. Except position 92, all these asymmetric positions are only in the bound PR2 and not in unbound PR2. Three of them (23, 31, and 46) are localized inside the binding pocket, and position 75 corresponds to a rigid position supposedly involved in long-range effects of ligand binding. Thus, our results suggest that these differences in asymmetry at positions 23, 31, 46, and 75 can play a role in the affinity differences of APV and DRV for PR2.

## Discussion

In this study, we focused on the analysis of local asymmetry—i.e., differences in backbone conformation—between the two monomers of the PR2 homodimeric protein to better understand the deformation and adaptation of PR2 to various ligands. It was previously shown that structural asymmetry, defined as differences in side-chain or main-chain conformations between two chains, can play a role in the capacity of a protein to recognize and bind different ligands (proteins, ligands, substrates or inhibitors) by inducing structural change in the binding site resulting in adaptive recognition^31,32^, in the differentiation of high and low-affinity binding sites^33^, and in the differentiation of active from non-active binding sites^34^. In these previous studies, the global structural asymmetry in homodimers was identified by computing the angle of rotation or the RMSD between superimposed monomers^12,14,31–36^. Some other studies focused on quantifying local structural asymmetry by computing and comparing residue angles^35^ or Cα-Cα distances^37^ in each monomer. In contrast to these approaches, we used an original, easy and quick method based on the HMM-SA structural alphabet to locate asymmetric positions. A structural asymmetric position was defined as a position exhibiting different local structural conformations, defined using the HMM-SA structural letters, between chains A and B in a PR2 dimer. HMM-SA, like other structural alphabets, has been previously used to describe protein structure and characterize backbone deformation upon protein-protein interactions^16,37^ but it was the first time that a structural alphabet (SA) was used to detect structural asymmetry. The detection of structural asymmetry using the SA-based approach consists of a simple comparison of letter sequences of two monomers. Contrary to other approaches, it does not require angle or distance computation and monomer superposition. However, the SA-based approach detects only structural deformation observed in the backbone, while the angle- and distance-based methods can be adapted to detect structural changes between two monomers observed in side chains.

**Figure 7:**
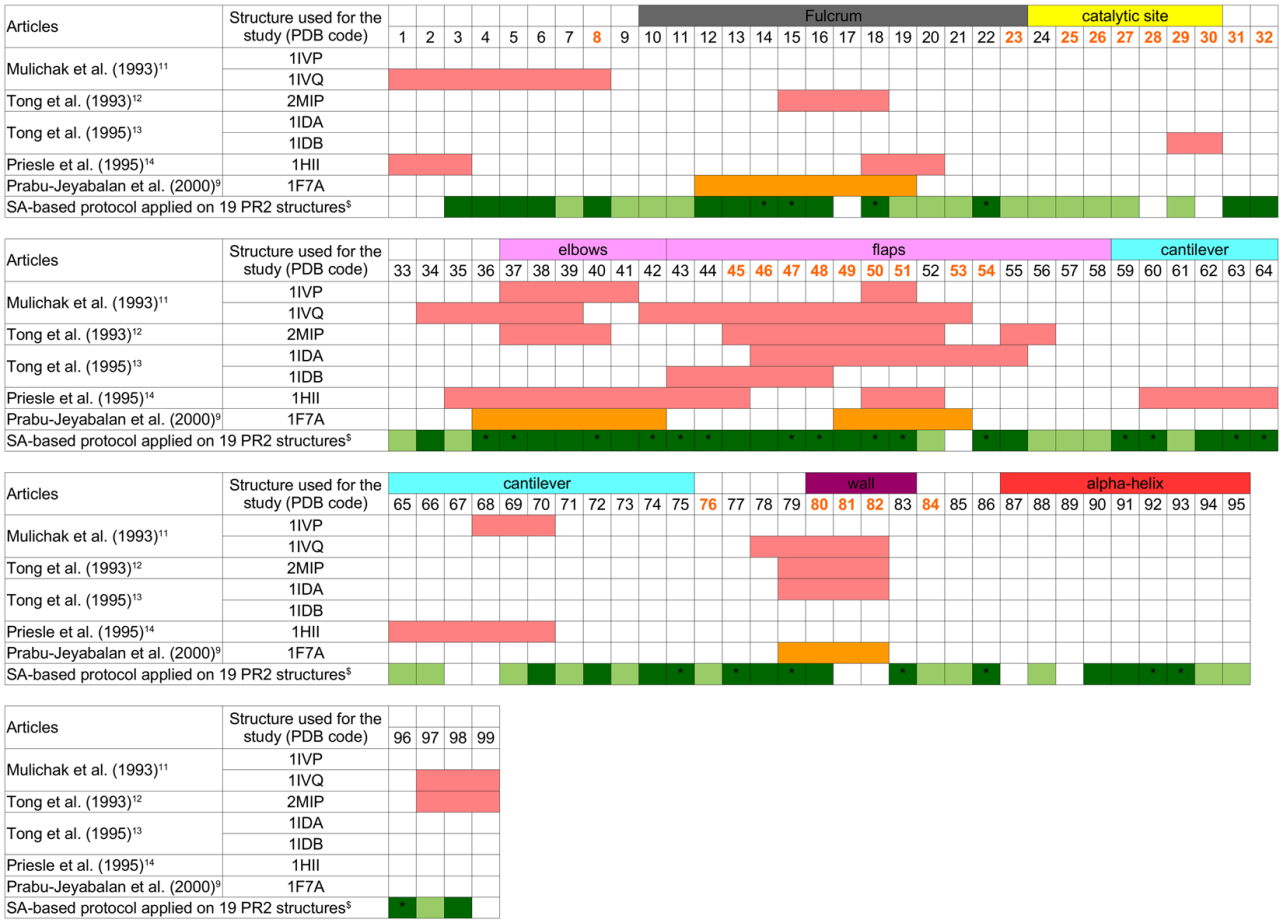
Comparison of highlighted structural asymmetric regions using the SA-based protocol with literature. In salmon are presented asymmetric positions in PR2 from literature^11–14^. In orange are presented asymmetric positions in PR1 from literature^9^. In green are presented asymmetric positions in PR2 detected using the SA-based protocol and the set of 19 PR2 structures. Light green highlights positions detected as asymmetric in less than five PR2 structures (*AO* < 5) using the SA-based protocol. Dark green highlights positions detected as asymmetric in at least five PR2 structures (*AO* ≥ 5) using the SA-based protocol. The symbol “*” indicates asymmetric position detected as overrepresented using the SA-based protocol. Structural and functional regions are indicated using coloured rectangles above residue numbers. Residue numbers coloured in orange highlight pocket residues. ^$^The 19 PR2 structures used for this analysis are listed in Table S1.

We applied the SA-based protocol to localize and quantify the backbone asymmetry on a set of 19 PR2 structures. We demonstrated that PR2 presents large structural backbone asymmetries with 90% of positions asymmetric in at least one PR2 structure. These asymmetric positions are distributed all along the structure, particularly in tail regions (3–6 and 96–98), the β-strand of the flap regions (42–47) or fulcrum (12–16), outer loops (elbow: 36–42, flap loop: 47–52, and residues 77–80), and the α-helix (90–93). By comparing the existing literature, most of these regions have been previously detected as asymmetric in several PR2 complexes^11–14^, as illustrated by Fig. 7. For example, by computing the RMSD between residues of the two monomers in a PR2 structure complexed with a peptide (PDB code: 2MIP), Tong *et al*. (1993) showed that this PR2 structure exhibits structural asymmetry in regions 15–18, 37–40, 45–52, and 78–82^12^. Seven positions (17, 30, 53, 67–68, and 81–82) are detected as asymmetric in some PR2 complexes^11,14^ but not with the SA-based protocol (Fig. 7). All these positions exhibit loop structural letters. Among the 27 structural letters of HMM-SA, 18 describe protein loops. Some structural letters dedicated to loop characterization are associated with a relatively large variability, such as structural letters *F* and *J* (observed at positions 17, 68, and 53). They are less accurate than those used to describe the local conformation of regular secondary structures (helices and strands), in agreement with the structural diversity of protein loops^20^. These loop structural letters probably include small structural changes inside the same letter. Thus, these structural letters are probably not sufficiently accurate to capture small structural changes detected using RMSD. By contrast, structural letters dedicated to regular secondary structures are very accurate, particularly those describing helices. Thus, the SA-based approach is the only method that detects backbone structural asymmetry in the α-helix (positions 90–91). This structural variability of the α-helix region can also be explained by the ability of the SA-based protocol to analyse a set of 19 PR2 structures complexed with various ligands (instead of a single PR2 complex in other studies), considering the ligand diversity and allowing us to highlight new asymmetric regions. In addition, this shows that HMM-SA is an efficient tool to precisely describe helices and that the SA-based protocol is effective in extracting structural asymmetry even in conserved regions.

Analysis of the structural asymmetry of PR2 using a large and diverse set of structures allowed us to distinguish between two types of asymmetry: ligand specific and recurrent structural asymmetry in PR2 dimers. Our results showed that 28% of PR2 positions are statistically overrepresented in the PR2 set. These asymmetric positions are conserved across the different PR2 dimers regardless of their form (unbound or bound), the different co-crystallized ligands, and the different experimental conditions. These asymmetries were detected in both the flexible and rigid positions, suggesting several explanations. The structural asymmetry occurring at flexible positions could result from the intrinsic flexibility of these positions, thus corresponding to an intrinsic property of the PR2. These asymmetric positions are observed along the whole PR2 sequence and structure, with a strong concentration in the flexible positions of the elbow, beginning of the flap, and the cantilever. The overrepresentation of asymmetry in the elbow and flap regions agrees with the structural asymmetry observed in several crystallographic structures^11–14^ but some have not been detected before (asymmetric positions in the α-helix) (Fig. 7).

The PR2 dimer undergoes large structural changes upon ligand binding, particularly in the elbow and flap regions, allowing the transition from the semi-open (unbound dimer) to closed (bound dimer) conformations, as illustrated by molecular dynamics simulations of PR2 complexed with APV and DRV^38,39^. In previous studies, it has been shown that local asymmetry is an intrinsic influence for the target to adopt different conformations^40,41^, particularly in the PR of HIV-1^10^. According to these observations, we suppose that the structural asymmetry linked to the flexibility of PR2, particularly in the elbow and beginning of the flaps, may be involved in the structural changes of the dimer and, thus, in the transition between the two main conformations. Our results showed that the overrepresented asymmetric and flexible position 40 showed a particular behaviour because it has a larger magnitude in most bound forms than in unbound PR2. Chen *et al*. (2014) showed that residue 40 of both chains of PR2 complexed with APV and DRV exhibits different dynamic behaviours using molecular dynamics simulations^38^. These results suggest that this residue exhibits an intrinsic asymmetry that increases upon ligand binding, even if it is outside of the binding pocket. Thus, we suggest that residue 40, located in the elbow, may play an important role in the structural changes induced on the flaps upon ligand binding in a cooperative mechanism between residues inside and outside of the binding pocket to induce flap movement. Other overrepresented asymmetric positions correspond to rigid positions. The structural asymmetry of these residues can result from crystal packing or the flexibility of neighbouring residues. Indeed, it is important to consider that the structural asymmetry of each PR2 dimer was detected by comparing the structural-letter sequence of the two chains. A structural letter denotes the geometry of 4-Cα fragments and determines the local conformation of a residue by considering its neighbourhood.

Our analyses also highlighted the structural asymmetry that may result from PR2 dimerization. All interface residues were asymmetric in at least one PR2 dimer, with an average of 30% of the interface residues of a PR2 dimer exhibiting structural asymmetry. Two interface positions (50 and 51) were detected as asymmetric in all PR2 dimers, in agreement with previous observations^11,40^. Interestingly, the asymmetry magnitude of these residues was larger in the unbound than in the bound dimers. This difference is linked to a decrease in their flexibility and could be explained by a hydrogen bond mediated by a water molecule between these two residues in the bound dimer only^14^. The asymmetry of other positions located in the interface seems to be mainly affected by specific ligand binding as identified in only a few of the bound structures. This is expected because of the location of the ligand-binding pocket at the PR2 interface between the two monomers.

Our results showed that, on average, a pocket contains 31% (±9) asymmetric positions, indicating that 80% of positions of the consensus pocket (24 residues seen in at least one PR2 pocket) are asymmetric and 5 residues are non-asymmetric (28, 30, 53, 81, and 82). Positions 28, 30, and 82 have been previously highlighted as interacting directly with ligands^11,13,42,43^. Thus, we suppose that this symmetry is important to ligand binding, and their localization can help to identify interacting residues. In addition, these results confirm that the PR2 binding pocket contains two types of residues: residues that adjust their backbone conformation in response to ligand binding and those that can be important for ligand binding. According to our results, the pocket asymmetric residues are mainly located at regions 23–27, 29, 31–32, 45–51, 54, 80, and 84, suggesting that this backbone asymmetry could be involved in PR2 ligand recognition, as previously proposed by several studies for positions 29, 45–51, and 80–81^11,13^. For example, Mulichak *et al*. (1993) showed that the shift observed at regions 79–82 of chain B in PR2 complexed with a peptidic inhibitor (PDB code: 1IVQ) widens the pocket to accommodate the inhibitor^11^. Tong *et al*. (1995) showed that the structural backbone asymmetry at position 29 is involved in the recognition of a short inhibitor^13^. Fifty percent of these pocket positions are asymmetric in bound PR2 structures and are not in the unbound PR2. This suggests that ligand binding directly induces this structural asymmetry potentially involved in the ligand recognition. Concerning the bound-specific asymmetry located outside of the binding site, 46% of them correspond to rigid positions. The location, rigidity and unbound-PR2 specificity of these positions suggest that this structural asymmetry could result from indirect effects of ligand binding. Thus, the structural changes occurring in the binding site may be accompanied by changes in other regions, particularly in the α-helix, end of the flaps, and beginning of the fulcrum regions, underlying the cooperation between these regions in PR2 structural changes upon ligand binding. Some cooperation in the motions in PR2 has been previously observed. Indeed, using molecular dynamics simulations of PR1 and PR2 complexed with APV and DRV, Chen *et al*. (2014) showed correlated motions of residues 60–90 relative to residues 10–30 in both chains of PR1. These correlated motions are also observed in PR2 but with a smaller magnitude. This structural asymmetry may also result from crystal packing or certain experimental conditions such as the crystallographic space group or, more likely, a coupling of the space group, the resolution and the co-crystallized ligand. The structural asymmetry observed at positions 16–19, 37–41, and 68–70 has been previously characterized as resulted from crystal packing interactions^11,12^. That some asymmetric positions are detected only in bound PR2 structures must be considered with caution, as the PR2 set contains only one unbound PR2 structure, preventing us from assessing the robustness of the results. However, as the unbound PR2 dimer is crystallized in the same space group as some bound PR2 structures, we considered that this unbound PR2 structure is representative for our analysis.

Our results suggest that numerous pocket asymmetric positions are specific to some PR2 structures. For example, positions 8, 18, and 84 seem to be involved in the specific recognition of ligands derived from 4-hydroxycoumarin (HETATM codes 3UPJ, 5UPJ, and 6UPJ), and positions 23, 31, and 32 may be important for the recognition of DRV and chemically related molecules such as APV (Tanimoto coefficient = 0.92 ± 0.02). Our results showed that the binding pocket of PR2 exhibits different structural asymmetry according to the bound ligand and that PR2 can adjust to various ligands. This allows PR2 to exhibit its wide range of ligand specificity. Thus, the detection of an asymmetric position specific for a given ligand type could provide information about the specific recognition of this ligand type and the mechanism of PR2 adaptation to this ligand type. In addition, it has been shown that local structural asymmetry in the binding site may play a role in ligand affinity^41^. For example, Jin *et al*. (1999) detected a structural asymmetry adjacent to the nucleotide-binding sites of aspartate transcarbamoylase that would appear to account for their separation into high- and low-affinity binding sites^33^. Despite their strong chemical similarity (Tanimoto coefficient = 0.95), DRV and APV exhibit completely different susceptibility against PR2^2,7^. By comparing the asymmetric profiles of PR2 dimers complexed with APV and DRV, we observed some differences that may be involved in the better recognition or higher affinity between DRV and PR2, particularly in the binding pockets at positions 23, 31, 46, and 75. To study this point in more detail, deeper analysis using molecular dynamics simulations is required.

Several studies have focused on the analysis of the structural asymmetry of PR1 structures. Prabu-Jeyabalan *et al*. (2000) analysed the asymmetry of PR1 in relation to its substrate binding, and they demonstrated that substrate binding breaks PR1’s symmetry^9^. They concluded that this structural asymmetry is important for the ligand recognition because it induces in PR1 conformational change of its monomer to adapt to the various ligands that are non-symmetric. Comparison of PR1 and PR2 structural asymmetry could provide information to better understand the PR2 resistance to PIs. Priestle *et al*. (1995) showed that the PR1 and PR2 structures complexed with the CGP 53820 inhibitor exhibit slightly different orientations of their two monomers and that the PR2 structure seems to fulfil the two-fold symmetry conditions better^14^. By comparing the conformation of the two monomers of PR1 complexed with a substrate, Prabu-Jeyabalan *et al*. (2000) showed that the most asymmetric PR1 regions were regions 12–19, 36–42, 49–53, and 79–82. The comparison of structural asymmetric positions detected in PR1 and PR2 targets showed that these regions are structurally asymmetric in PR1 and PR2 (Fig. 7). Priestle *et al*. (1995) revealed that region 36–44 is the most asymmetric in PR1 and PR2^12^. In their study, Jeyabalan *et al*. (2000) showed that region 25–29 exhibits the same conformation in both chains of PR1. In our study, we observed that residue 29 is asymmetric in PR2 and could have a role in the ligand recognition, in agreement with Tong *et al*. (1995)^12^. In addition, several comparisons of PR1 and PR2 complexes showed that regions 15–20 and 78–85 are more asymmetric in PR1 than in PR2^12,14^. In this work, residues 81 and 82 are detected as non-asymmetric in the PR2 set, in agreement with the weak asymmetry of regions 15–20 and 78–85 in PR2 previously detected in three structures (PDB codes: 1IVP, 1IDB, and 1HII)^11–13^. Prabu-Jeyabalan *et al*. (2000) showed that in PR1, the region 79–82 is important for ligand recognition and adaptation of the pocket to the substrate shape. Interestingly, residue 82 is substituted between PR1 and PR2 sequences (V82I) and is associated with the multi-drug resistance of HIV-1 to PIs^43^. These results suggest that this substitution could modify the residue flexibility, which has an impact on its capacity to move to adapt the PR2 pocket to the ligand’s shape. However, this result must be considered with caution because residues 81 and 82 are detected as asymmetric in three structures (PDB codes: 1IVQ, 1IDA and 2MIP)^11,13^. In addition, Tong *et al*. (1995) suggested that the asymmetry of positions 79–82 is important to the binding of a large ligand. To better understand the putative role of residue 82 in the resistance of PIs against PR2, it would be interesting to build PR structures with a mutation at position 82 and compare the wild-type and mutant asymmetric profiles. Analysing and understanding in more detail the differences between PR1 and PR2 in terms of ligand recognition are important to clarifying the PI resistance of PR2 and can allow the interpretation of these differences at the molecular level, facilitating the design of inhibitors that are equipotent with respect to both PRs.

## Conclusion

In summary, we have used an original tool based on HMM-SA to detect the asymmetric positions in all available PR2 dimers and to explain their important role in the changing behaviour of the monomers due to ligand binding. First, on average 31% of PR2 positions are asymmetric. These asymmetric positions are distributed all along the structure, particularly in terminal regions (3–6 and 96–98), β-strand of the flap regions (42–47) or fulcrum (12–16), outer loops (elbow: 36–42, flap loop: 47–52, and residues 77–80), and α-helix (90–93). It is the first time that the structural asymmetry of the α-helix of PR2 is highlighted. From these structural asymmetric positions, we differentiated overrepresented asymmetric positions (28%), which are also characterized by structural asymmetry of high magnitude, from the asymmetric positions with a low frequency (72%). Our results indicate that most of the structural asymmetric positions detected in the PR2 dimers are not explained by experimental conditions. Indeed, we showed that some of the detected asymmetries are intrinsic properties of PR2 and are induced by the flexibility of PR2. We suggest that this asymmetry, especially at position 40, is important for structural changes of PR2 and particularly for the transition from semi-open to closed conformations. Our results showed that ligand binding is also responsible for structural asymmetry in PR2. Most of these asymmetric positions are specific to some ligand types and seem to be important for PR2 structural changes allowing the adaptation of PR2 to various ligands and thus involved in the PR2’s recognition of various ligand. This structural asymmetry is accompanied by asymmetry located outside of binding site that suggests cooperation in the PR2 structural changes in different PR2 regions upon ligand binding. In addition, to better understand the different susceptibilities against PR2 of APV and DRV, two chemical similarity approved drugs, we compared their asymmetric profiles. We observed some differences, particularly in the binding pockets at positions 23, 31, 46, and 75. We suggest that these positions might be involved in the better recognition or higher affinity between DRV and PR2 relatively to APV. To conclude, our study provides for the first time a large and robust description and characterization of PR2 asymmetry in both bound and unbound structures. Our results provide new insights about PR2 structures allowing a better understanding of the structural changes of PR2 and its diverse ligand recognition. Understanding and taking advantage of such conformational flexibility will be important in the design and optimization of HIV-2 protease inhibitors of HIV-2 protease. They provide some new insights explaining the low sensitivity of PR2 to commercially available protease inhibitors, paving the way to deeper analysis about the role of these asymmetric positions and the ligand characteristics required for strong efficiency against HIV-2.

To improve the study of the link between the structural asymmetry of PR2 and its ligand recognition and specificity, it will be interesting to characterize more precisely the structural asymmetry. One way to do so could be to characterize the transition of structural changes for each position—i.e., the two structural letters in the two chains. This description will allow the structural asymmetry to be classified and studied in more detail if different classes of structural asymmetry exist and their link between ligand types. In a next step, the SA-based protocol could be applied to PR2 structures generated using molecular dynamics simulations of PR2 in unbound and bound forms that could allow studying PR2 asymmetry in relation with its dynamic behaviour and confirming the putative role of some asymmetric residues in ligand recognition.

## Electronic supplementary material

Supplementary Information

## References

[1] Visseaux B, Damond F, Matheron S, Descamps D, Charpentier C (2016). Hiv-2 molecular epidemiology. Infect. Genet. Evol..

[2] Raugi DN, Smith RA, Gottlieb GS (2016). University of Washington-Dakar HIV-2 Study Group. Four Amino Acid Changes in HIV-2 Protease Confer Class-Wide Sensitivity to Protease Inhibitors. J. Virol..

[3] Desbois D (2008). *In vitro* phenotypic susceptibility of human immunodeficiency virus type 2 clinical isolates to protease inhibitors. Antimicrob. Agents Chemother..

[4] Masse S (2007). *In vitro* selection and characterization of human immunodeficiency virus type 2 with decreased susceptibility to lopinavir. Antimicrob. Agents Chemother..

[5] Bénard A (2009). Good response to lopinavir/ritonavir-containing antiretroviral regimens in antiretroviral-naive HIV-2-infected patients. AIDS.

[6] Rodés B (2006). Susceptibility to protease inhibitors in HIV-2 primary isolates from patients failing antiretroviral therapy. J. Antimicrob. Chemother..

[7] Brower ET, Bacha UM, Kawasaki Y, Freire E (2008). Inhibition of HIV-2 protease by HIV-1 protease inhibitors in clinical use. Chem. Biol. Drug Des..

[8] Ntemgwa ML (2009). Nucleoside and nucleotide analogs select in culture for different patterns of drug resistance in human immunodeficiency virus types 1 and 2. Antimicrob Agents Chemother..

[9] Prabu-Jeyabalan M, Nalivaika E, Schiffer CA (2000). How does a symmetric dimer recognize an asymmetric substrate? A substrate complex of HIV-1 protease. J. Mol. Biol..

[10] Prabu-jeyabalan M (2003). Viability of a drug-resistant human immunodeficiency virus type 1 protease variant: structural insights for better antiviral therapy. Society.

[11] Mulichak AM (1993). The crystallographic structure of the protease from human immunodeficiency virus type 2 with two synthetic peptidic transition state analog inhibitors. J. Biol. Chem..

[12] Tong L (1993). Crystal structure of human immunodeficiency virus (HIV) type 2 protease in complex with a reduced amide inhibitor and comparison with HIV-1 protease structures. Proc. Natl. Acad. Sci. USA.

[13] Tong L (1995). Crystal structures of HIV-2 protease in complex with inhibitors containing the hydroxyethylamine dipeptide isostere. Structure.

[14] Priestle JP (1995). Comparative analysis of the X-ray structures of HIV-1 and HIV-2 proteases in complex with CGP 53820, a novel pseudosymmetric inhibitor. Structure.

[15] Camproux A-C, Gautier R, Tufféry P (2004). A hidden Markov model derived structural alphabet for proteins. J. Mol. Biol..

[16] Martin J, Regad L, Lecornet H, Camproux A-C (2008). Structural deformation upon protein-protein interaction: a structural alphabet approach. BMC Struct. Biol..

[17] Regad L, Martin J, Camproux A-C (2011). Dissecting protein loops with a statistical scalpel suggests a functional implication of some structural motifs. BMC Bioinformatics.

[18] Berman HM (2000). The Protein Data Bank. Nucleic Acids Res..

[19] Regad L, Guyon F, Maupetit J, Tufféry P, Camproux A-C (2008). A Hidden Markov Model applied to the protein 3D structure analysis. Comput. Stat. Data Anal..

[20] Regad L, Martin J, Nuel G, Camproux A-C (2010). Mining protein loops using a structural alphabet and statistical exceptionality. BMC Bioinformatics.

[21] Pérot S (2013). Insights into an original pocket-ligand pair classification: A promising tool for ligand profile prediction. PLoS One.

[22] O’Boyle NM (2011). Open Babel: An Open chemical toolbox. J. Cheminform..

[23] R Development Core Team, in R: A language and environment for statistical computing, Vienna, Austria,, http:// www.R-project.org/ (2013).

[24] Sadiq SK, de Fabritiis G (2010). Explicit solvent dynamics and energetics of HIV-1 protease flap opening and closing. Proteins Struct. Funct. Bioinforma..

[25] De Beer TAP, Berka K, Thornton JM, Laskowski RA (2014). PDBsum additions. Nucleic Acids Res..

[26] Borrel A, Regad L, Xhaard H, Petitjean M, Camproux A-C (2015). PockDrug: A Model for Predicting Pocket Druggability That Overcomes Pocket Estimation Uncertainties. J. Chem. Inf. Model..

[27] Caumes, G., Borrel, A., AbiHussein, H., Camproux, A.-C. & Regad, L. Investigating the Importance of the Pocket-estimation Method in Pocket-based Approaches: An Illustration Using Pocket-ligand Classification. *Mol. Inform*. **36**, 10.1002/minf.201700025 (2017). 10.1002/minf.201700025 28452177

[28] Cerisier, N., Regad, L., Triki, D., Camproux, A.-C. & Petitjean, M. Cavity Versus Ligand Shape Descriptors: Application to Urokinase Binding Pockets. *J. Comput. Biol*. **36**, 10.1002/minf.201700040 (2017). 10.1089/cmb.2017.0061 PMC5684670 28570103

[29] Karplus PA, Schulz GE (1985). Prediction of chain flexibility in proteins. Naturwissenschaften.

[30] Parthasarathy S, Murthy MR (1997). Analysis of temperature factor distribution in high-resolution protein structures. Protein Sci..

[31] Xiao B (1999). Crystal structure of a murine glutathione S-transferase in complex with a glutathione conjugate of 4-hydroxynon, 2-enal in one subunit and glutathione in the other: Evidence of signaling across the dimer interface. Biochemistry.

[32] Cha H, Kopetzki E, Huber R, Lanzendörfer M, Brandstetter H (2002). Structural basis of the adaptive molecular recognition by MMP9. J. Mol. Biol..

[33] Jin L, Stec B, Lipscomb WN, Kantrowitz ER (1999). Insights into the mechanisms of catalysis and heterotropic regulation of Escherichia coli aspartate transcarbamoylase based upon a structure of the enzyme complexed with the bisubstrate analogue N-phosphonacetyl-L-aspartate at 2.1 Å. Proteins Struct. Funct. Genet..

[34] Renatus M, Stennicke HR, Scott FL, Liddington RC, Salvesen GS (2001). Dimer formation drives the activation of the cell death protease caspase 9. Proc. Natl. Acad. Sci. USA.

[35] Pednekar D, Durani S (2010). Protein homomers in point-group assembly: Symmetry making and breaking are specific and distinctive in their codes of chemical alphabet in side chains. Proteins Struct. Funct. Bioinforma..

[36] King DA, Zhang L, Guarente L, Marmorstein R (1999). Structure of a HAP1-DNA complex reveals dramatically asymmetric DNA binding by a homodimeric protein. Nat. Struct. Biol..

[37] Swapna LS, Srikeerthana K, Srinivasan N (2012). Extent of structural asymmetry in homodimeric proteins: Prevalence and relevance. PLoS One.

[38] Kar P, Knecht VJ (2012). Origin of decrease in potency of darunavir and two related antiviral inhibitors against HIV-2 compared to HIV-1 protease. Phys Chem B..

[39] Chen J (2014). Revealing origin of decrease in potency of darunavir and amprenavir against HIV-2 relative to HIV-1 protease by molecular dynamics simulations. Sci Rep..

[40] Goodsell DS, Olson AJ (2000). Structural symmetry and protein function. Annu. Rev. Biophys. Biomol. Struct..

[41] Brown JH (2006). Breaking symmetry in protein dimers: designs and functions. Protein Sci..

[42] Tie, Y. *et al*. Critical differences in HIV-1 and HIV-2 protease specificity for clinical inhibitors. **21**, 339–350 (2012). 10.1002/pro.2019 PMC3375435 22238126

[43] Kovalevsky AY, Louis JM, Aniana A, Ghosh AK, Weber IT (2008). Structural evidence for effectiveness of darunavir and two related antiviral inhibitors against HIV-2 protease. J Mol Biol..

