# Supplementary material for: Analysis of the HIV-2 protease’s adaptation to various ligands: characterization of backbone asymmetry using a structural alphabet
Source: Sci Rep. 2018 Jan 15;8:710. doi: 10.1038/s41598-017-18941-3 (PMC5768731; doi:10.1038/s41598-017-18941-3)
Supplement: Supplementary file 1 — Supplementary Information [file 41598_2017_18941_MOESM1_ESM.pdf]

Supplementary Information

TITLE : Analysis of the HIV-2 protease's adaptation to various ligands: characterization of backbone asymmetry using a structural alphabet

Authors: Dhoha Triki[1,3], Mario Enrique Cano Contreras[1,3], Delphine Flatters[1,3], Benoit Visseaux[2,3], Diane Descamps[2,3], Anne-Claude Camproux[1,3] & Leslie Regad[1,3,*].

[1]Molécules thérapeutiques *in silico* (MT*i*), INSERM UMR-S973, Paris, France

[2]IAME, UMR 1137, INSERM, Laboratoire de Virologie, Hôpital Bichat, AP-HP, Paris, France

[3]Université Paris Diderot, Sorbonne Paris Cité, Paris, France

* leslie.regad@univ-paris-diderot.fr

Supplementary Table S1: Presentation of the PR2 set. It presents descriptions of the 19 PDB files with their PDB code, corresponding X-ray space group, resolution and complexed ligand. Each ligand was named using the HETAM code (code extracted from the PDB file). Stars indicate the PR2 dimers that exhibit the experimental mutation K57L.

PDB code	Space Group	Resolution (Å)	Ligand (HETATM code)
1HSI	$P\ 1\ 2_1\ 1$	2.50	no
1HSH	$P\ 1\ 2_1\ 1$	1.90	IDV
2HPE*	$P\ 1\ 2_1\ 1$	2	Peptide
5UPJ*	$P\ 2_1\ 2_1\ 2_1$	2.30	UIN
3UPJ*	$P\ 2_1\ 2_1\ 2_1$	2.50	U03
6UPJ*	$P\ 2_1\ 2_1\ 2_1$	2.34	NIU
1IVP*	$P\ 2_1\ 2_1\ 2_1$	2.50	1ZK
1HII	$P\ 2_1\ 2_1\ 2_1$	2.3	C20
1IVQ*	$P\ 2_1\ 2_1\ 2_1$	2.60	0PX
4UPJ*	$P\ 2_1\ 2_1\ 2_1$	1.90	U04
1JLD	$P\ 4_3\ 2_1\ 2$	2.50	0PP
1IDA	$P\ 4_3\ 2_1\ 2$	1.70	0PO
1IDB	$P\ 4_3\ 2_1\ 2$	2.20	0DO
2MIP	$P\ 4_3$	2.20	Peptide
2HPF*	$P\ 6_5$	3	Peptide
3S45	C 1 2 1	1.51	APV
3ECG	C 1 2 1	1.18	065
3EBZ	C 1 2 1	1.20	DRV
3EC0	C 1 2 1	1.18	GRL

Supplementary Note 1: Computation of the overrepresentation of the structural asymmetric position

To evaluate whether structural asymmetry was observed frequently or rarely (due to a particular ligand or particular experimental condition) in the PR2 set, we computed, for each position, the number of PR2 dimers exhibiting the position as asymmetric, and we named this asymmetry occurrence (*AO)*. The statistical significance of the *AO* value of a position *i* was determined using the overrepresentation p-value of any *AO*, denoted $pvalue^{AO}$. The $pvalue^{AO}$ was computed by comparing the observed *AO* with the expected *AO* computed in a random set, denoted AO^{random}. More precisely, $pvalue^{AO}$ was estimated as the probability that $AO^{random}(i)$ is higher than *AO(i)* using a set of 2000 generated random sets (Equation 1):

$pvalue^{AO}(i) = p[AO^{random}(i) > AO(i) = n\{ AO^{random}(i) > AO(i)\} / n_{simu}$ Equation 1

where $n\{AO^{random}(i) > AO(i)\}$ is the number of simulations, $AO^{random}(i)$ is higher than *AO(i)*, and n_{simu} is the number of simulations.

Each random set has the same size as the PR2 set—i.e., composed of 19 random sequences of 96 positions—and each random sequence is associated with a PR2 dimer. Each random sequence corresponds to a binary sequence where each position takes two possible values, 0 and 1, representing symmetric and asymmetric positions, respectively. The generation of the random binary sequence corresponding to the jth PR2 dimer conserves the number of asymmetric positions in the jth PR2 dimer. The expected asymmetry occurrence of position *i* corresponds to the number of random sequences in which this position is considered an asymmetric position—i.e., with a value of 1 at this position.

An asymmetric position was considered statistically overrepresented if its $pvalue^{AO}$ was below a threshold of 0.0005 as determined using the Bonferroni adjustment to consider multiple tests (0.05/96 positions).

Supplementary Note 2: Determination of residues involved in the PR2 interface

The PR2 interface was determined by extracting residues with differences in terms of the accessible surface area (ASA) of more than 5 $Å^2$ in the PR2 in dimer form—i.e., in structures that contain the two monomers—and in monomer form—i.e., in structures that contain only one monomer. We first computed the ASA of all residues of each monomer in each dimer form, denoted ASA_{dim}. Second, we manually created the PR2 structures in the monomer form by splitting the two monomers into two different PDB files. For each PR2 in the monomer form, we then computed the ASA of all residues, denoted ASA_{mono}. ASA_{mono} and ASA_{dim} were computed using NACCESS software[1]. For residue *i* of PR2 structure *j*, we determined the change in ASA upon the dimerization of PR2, denoted $\Delta ASA(i,j)$, which corresponds to the difference between its ASA in the dimer and monomer forms of PR2 structure *j* (Equation 2).

$\Delta ASA(i,j) = ASA_{mono}(i,j) - ASA_{dim}(i,j)$ Equation 2

Residues with a $\Delta ASA(i,j)$ higher than 5 Å^2 were defined as being involved in the interface of the studied PR2 dimer. The global PR2 interface was finally defined as the 31 residues involved in the interface in at least 80% of all PR2 dimers (Fig. 3).

Supplementary Note 3: Identification of flexible and rigid positions

The rigid and flexible positions of PR2 were determined using normalized B-factor values[2] (temperature factor/atomic displacement factor, denoted B_{norm}) extracted from the PDB files. This B-factor value reflects the degree of isotropic smearing of the electron density around its centre[3]. However, B-factor values cannot be used directly because they may be on different scales owing to the application of different refinement procedures[36]. Therefore, we normalized them such that each PR2 structure would have a distribution with a mean of zero and unit variance. The normalized B-factor value[37] of residue *i* of PR2 *j* is computed using Equation 3:

$B_{norm}(i,j) = [B_{(i,j)} - < B_j >] / \sigma_{Bj}$ Equation 3

where B_i is the B-factor value of the Cα atom of residue *i*, taken from the PDB file of PR2 *j*, and $<B_j>$ and σ_{Bj} are the average value and standard deviation of the B-factor values of all Cα atoms of PR2 *j*. For a PR2 position, we then computed its average B_{norm} value using the B_{norm} values of residues at this position in the 19 PR2 structures. A flexible position is defined as a position with an average B_{norm} higher than 0 in either chain A or chain B or in both. A rigid position is defined as a position with an average B_{norm} smaller than 0 in both chains A and B. The comparison of the structural asymmetry of flexible and rigid positions, defined in terms of AO, was performed using Student test.

References

1. Hubbard SJ, Thornton JM (1993) NACCESS, Computer Program, Department of Biochemistry and Molecular Biology, University College London.
2. Karplus, P. A. & Schulz, G. E. Prediction of chain flexibility in proteins. Naturwissenschaften 72, 212–213 (1985).
3. Parthasarathy, S. & Murthy, M. R. Analysis of temperature factor distribution in high-resolution protein structures. Protein Sci. 6, 2561–2567 (1997).
